# Longitudinal immune profiling of a SARS-CoV-2 reinfection in a solid organ transplant recipient

**DOI:** 10.21203/rs.3.rs-405958/v1

**Published:** 2021-05-05

**Authors:** Jon Klein, Anderson Brito, Paul Trubin, Peiwen Lu, Patrick Wong, Tara Alpert, Mario Pena-Hernandez, Winston Haynes, Kathy Kamath, Feimei Liu, Chantal Vogels, Joseph Fauver, Carolina Lucas, Ji Eun Oh, Tianyang Mao, Julio Silva, Anne Wyllie, M. Catherine Muenker, Arnau Casanovas-Massana, Adam Moore, Mary Petrone, Chaney Kalinich, YALE IMPACT RESEARCH TEAM, Charles Dela Cruz, Shelli Farhadian, Aaron Ring, John Shon, Albert Ko, Nathan Grubaugh, Benjamin Goldman-Israelow, Akiko Iwasaki, Marwan Azar

**Affiliations:** Yale University; Yale; Yale University; Yale School of Public Health; Yale; Serimmune; Yale University School of Medicine; Yale School of Public Health; Yale University; Korea Advanced Institute of Science and Technology; Yale University; Yale School of Medicine; Yale School of Public Health; Yale University; Yale; Yale School of Public Health; Yale School of Medicine; Yale School of Public Health; Yale University School of Medicine; Yale University; Yale School of Medicine; Serimmune (United States); Yale School of Public Health; Yale University School of Medicine; Yale University; Yale

**Keywords:** SARS-CoV-2 reinfection, solid organ transplant recipient, immune profiling, pathogen adaptation

## Abstract

The underlying immunologic deficiencies enabling SARS-CoV-2 reinfections are currently unknown. Here we describe a renal-transplant recipient who developed recurrent, symptomatic SARS-CoV-2 infection 7 months after primary infection. To elucidate the immunological mechanisms responsible for reinfection, we performed longitudinal profiling of cellular and humoral responses during both primary and recurrent SARS-CoV-2 infection. We found that the patient responded to the primary infection with transient, poor-quality adaptive immune responses that was further compromised by intervening treatment for acute rejection of the renal allograft prior to reinfection. Importantly, we identified the development of neutralizing antibodies and humoral memory responses prior to SARS-CoV-2 reinfection. However, these neutralizing antibodies failed to confer protection against reinfection, suggesting that additional factors are required for efficient prevention of SARS-CoV-2 reinfection. Further, we found no evidence supporting viral evasion of primary adaptive immune responses, suggesting that susceptibility to reinfection may be determined by host factors rather than pathogen adaptation.

## Introduction

The dynamics and duration of adaptive immune responses to SARS-CoV-2 infection have been described in association with disease severity and the rate of viral clearance, yet the correlates of adaptive immunity responsible for preventing reinfection remain incompletely characterized. In studies of SARS-CoV-2 infection in animal models (mice^1,2^, hamsters^3,4^, and rhesus macaques^5–8^), both vaccine-induced and natural infection-induced immunity are sufficient for protection from SARS-CoV-2 rechallenge. Recent Phase III vaccine clinical trials^9^, as well as epidemiologic studies of natural infection^10^, have also demonstrated robust development of protective immunity in humans. Taken together, these data unambiguously demonstrate that adaptive immunity confers protection against SARS-CoV-2 infection in the majority of cases. However, rare case reports of SARS-CoV-2 reinfection by antigenically similar variants have also been documented as soon as 48 days from primary symptom onset^11–18^ (Extended Data Table 1). Whether these reinfections are the direct result of deficient adaptive immune responses to the primary infection, or are the result of waning adaptive immunity, is currently unknown.

Notably, cases of persistent SARS-CoV-2 infection among patients with underlying genetic defects (such as X-linked agammaglobulinemia^19^) or acquired defects (B cell depletion therapy^20^) in humoral immunity have been reported. Immunocompromised COVID-19 patients with prolonged infection also achieved viral clearance when treated with multiple doses of convalescent plasma^21^, demonstrating the sufficiency of humoral responses in clearing SARS-CoV-2 infection. The role of cellular immunity in protection from SARS-CoV-2 infections is also a subject of intense investigation. Studies demonstrate that most COVID-19 patients develop SARS-CoV-2 specific CD4^+^ and CD8^+^ T cells^22^ and reports of durable T-cell memory responses to related SARS-CoV-1 infection lasting up to 17 years after initial infection^23^. While neutralizing antibodies are a correlate of protection, non-human primate models have demonstrated reduced virological control in the upper respiratory tract in CD8^+^ depleted convalescent animals upon reinfection^24^, suggesting that both arms of the adaptive immune response may be required for optimal clearance and protection against SARS-CoV-2 infection.

Due to the rarity and complexity involved in investigation of human SARS-CoV-2 reinfections, complete immune profiles exploring the magnitude and extent of these adaptive immune responses in paired primary infection and reinfection are lacking. Identifying the deficient features of initial adaptive immune responses that enables subsequent SARS-CoV-2 reinfection will help to further define the correlates of immune protection in humans.

## Results

### Clinical presentation of immunocompromised solid organ transplant recipient with SARS-CoV-2 reinfection.

**Figure 1:**
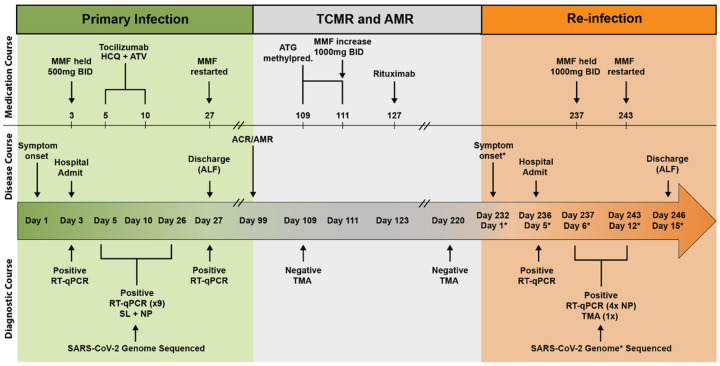
SARS-CoV-2 reinfection clinical timeline. Summary of patient’s disease course divided into distinct clinical periods: primary infection (green), graft rejection and immunosuppressive therapies (grey), and SARS-CoV-2 reinfection (orange). Clinical annotations are stratified into rows based on content (Medications, Disease Course, Diagnostic Testing). Arrows indicate specific events, brackets indicate duration of treatment or testing where applicable. Asterisks (*) indicate annotations specific to SARS-CoV-2 reinfection. Double line breaks ( / / ) indicate condensing of clinical timeline for display. Abbreviations (Top to bottom, left to right): T cell mediated rejection (TCMR); antibody-mediated rejection (AMR); mycophenolate mofetil (MMF); BID (twice daily); hydroxychloroquine (HCQ); atazanavir (ATV); anti-thymocyte globulin (ATG); methylprednisolone (methylpred.); assisted living facility (ALF); Real-time quantitative polymerase chain reaction (RT-qPCR); saliva (SL); nasopharyngeal (NP); transcription mediated amplification (TMA).

In March 2020, a 66-year-old man residing in a transitional group living facility with a medical history notable for bipolar disorder and end-stage renal disease due to lithium toxicity, for which he had undergone living-donor renal transplantation two years prior, was hospitalized with fevers, fatigue, and dry cough (Fig. 1). Induction immunosuppression for renal transplantation had consisted of antithymocyte globulin, while maintenance immunosuppression initially included tacrolimus (a calcineurin inhibitor that inhibits T-cell cytokine production), mycophenolate mofetil (MMF, a B and T lymphocyte anti-proliferative agent), and low-dose prednisone. By the time of hospitalization, belatacept (a T lymphocyte costimulation blocker) had been substituted for tacrolimus due to the development of calcineurin-induced neurotoxicity, and prednisone had been discontinued due to perceived exacerbation of psychiatric illness. Persistent neutropenia complicated the post-transplantation course, requiring substitution of prophylactic inhaled pentamidine for trimethoprim-sulfamethoxazole and frequent infusions of filgrastim. Upon hospitalization, SARS-CoV-2 infection was diagnosed via reverse-transcriptase polymerase chain reaction (RT-PCR) performed on a nasopharyngeal swab (NP) specimen. He was subsequently enrolled in the Yale Implementing Medical and Public Health Action Against Coronavirus CT (IMPACT) study, a biospecimen repository housing clinical and demographic data as well as respiratory, blood, and other tissue samples from patients with confirmed COVID-19 at Yale New Haven Hospital. He developed symptomatic moderate COVID-19 for which he received hydroxychloroquine and atazanavir for 5 days and a single dose of tocilizumab at 8 milligrams/kilogram (mg/kg). MMF was paused and a reduced dose of belatacept was administered in the setting of acute infection. The oxygen requirement peaked at 4 liters per minute by nasal cannula; by 13 days from symptom onset (DFSO), the patient was transitioned to room air. Though the patient was asymptomatic thereafter, nasopharyngeal (NP) swabs and saliva (SL) from the patient remained positive for SARS-CoV-2 by PCR throughout the hospital stay (Extended Data Table S2). The patient was discharged from the hospital on 27 DFSO to the transitional group residential facility after a 14-day period without hypoxia, reemergence of symptoms, or other clinical signs of infection. MMF was restarted on discharge.

Approximately 10 weeks after discharge, a kidney allograft biopsy was performed because of increasing serum creatinine and was notable for evidence of acute T-cell-mediated rejection (TMCR) and antibody-mediated rejection (AMR) of the transplanted organ (Fig. 1). He was readmitted and treated with 400 mg of antithymocyte globulin and 125 mg of methylprednisolone. Belatacept was continued, low-dose prednisone was restarted, and the MMF dose was increased. Notably, a nasopharyngeal swab collected at the time was negative for SARS-CoV-2 using a non-quantitative transcription-mediated amplification (TMA) test. The patient remained asymptomatic and was discharged back to the transitional living facility. He received rituximab 1 week after discharge to address AMR.

Approximately 15 weeks after this hospitalization, the patient underwent repeat renal allograft biopsy for evaluation of polyomavirus-associated nephropathy that demonstrated evidence of mild remnant AMR (Fig. 1). Ongoing neutropenia necessitated additional infusions of filgrastim. At 220 DFSO, a NP swab collected from the patient was again negative for SARS-CoV-2 using TMA.

Approximately 4 months after the diagnosis of rejection and 7 months from his primary COVID-19 diagnosis, the patient was readmitted to the hospital with fatigue and nonproductive cough (Fig. 1). Repeat SARS-CoV-2 PCR of NP samples returned positive at 236 DFSO / 5 days from reinfection symptom onset (DFSO*) with cycle thresholds to targets N1 and N2 of 27.34 and 27.15, respectively. The patient did not develop fevers or hypoxia, had no evidence of pneumonia on chest imaging, and did not require COVID-19-specific therapy. SARS-CoV-2 IgG was reactive at 5 DFSO*. Isolation precautions were reinstituted for the 10-day duration of hospitalization and were maintained after his return to the group living facility.

### Genome sequencing reveals two distinct lineages of SARS-CoV-2 during primary infection and reinfection

**Figure 2:**
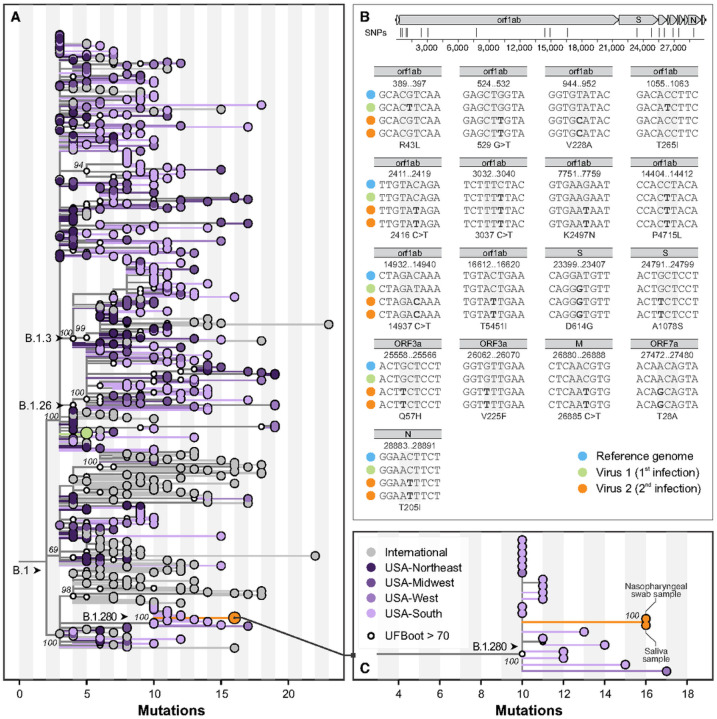
Maximum likelihood phylogeny of SARS-CoV-2 whole genomes. (A) Global tree showing the evolutionary relationship of 561 lineage B.1 SARS-CoV-2 genomes, including three samples from the patient’s two independent infections as described in this study (green, primary infection; orange, reinfection). These viruses belong to two sublineages, which evolved independently of each other since their most recent common ancestors, which circulated in the Northeast United States in March 2020. (B) Profile of mutations observed in genomes from both infections, compared with the reference genome (GenBank: MN908947), shown at the top, highlighting the positions of the SNPs shown in the panel. Highlights of the two genomic sequences obtained from the reinfection are shown. (C) A zoomed view of the clade demonstrating relatedness of viruses in the reinfection group reveals their relatedness to viruses that circulated in the Southern United States (state of Florida), the likely origin of that sublineage.

Following symptom onset during the primary infection in March 2020, both nasopharyngeal and saliva specimens tested positive by PCR, and nasopharyngeal specimens were whole genome sequenced for phylogenetic analysis^25^. Additional nasopharyngeal and saliva specimens were collected and sequenced during the reinfection episode in November 2020 (Extended Data Table 2). To rule out the possibility of persistent SARS-CoV-2 infection, which has been previously reported^26–29^, we compared the virus genomes sequenced from specimens collected 7 DFSO in the primary infection (NP swab), and 5 DFSO* during the reinfection (NP swab and saliva). Phylogenetic analysis revealed that viruses from the primary infection and reinfection belong to 2 distinct clades within the SARS-CoV-2 lineage B: clade B.1 in the primary infection in March 2020, and B.1.280 in the reinfection in November 2020 (Fig. 2a). Specifically, the virus genome sequenced from the reinfection (Fig. 2a, c **(green)**) had 12 mutations not observed in the virus sequenced from the primary infection (Fig. 2c **(orange)**): 4 synonymous and 8 non-synonymous. Among the mutations that alter amino acid identity relative to the SARS-CoV-2 reference genome (Wuhan- Hu-1, GenBank: MN908947), both viruses expressed the spike protein with glycine in position 614 (D614G), but only the virus from the reinfection had an additional polymorphism at spike A1078S, close to the transmembrane connector domain in the S2 subunit^30^ (Fig. 2b; Extended Data Fig. S2). Importantly this mutation is not located within the SARS-CoV-2 spike receptor binding domain, which is the primary target of neutralizing antibodies (Extended Data Fig. S2), nor has it been reported among SARS-CoV-2 variants of concern (VOC) B.1.1.7, B.1.351, or P1 that display variable evasion of humoral immune responses^31^.

Our phylogenetic analysis also demonstrates that the distinct viral lineages identified from the patient’s primary infection and subsequent reinfection diverged from their common ancestor around March 2020 (Fig. S1), suggesting intra-host evolution in the setting of persistent infection to be an unlikely explanation for this case and providing unambiguous evidence of reinfection. To rule out the remote possibility of the presence of multiple SARS-CoV-2 lineages during reinfection, we also sequenced virus genomes from both saliva and nasopharyngeal swabs collected during the reinfection (Fig. 1c) and found them to be identical. Lastly, we analyzed the geographic distributions of circulating SARS-CoV-2 lineages and discovered that the sub-lineage of viruses identified in the reinfection likely first circulated in the Southern US in June of 2020 before being reintroduced to the Northeast US. This patient’s primary residence is located within the Northeast, and he reported no travel since discharge from the primary SARS-CoV-2 infection in March of 2020 (Fig. S1), confirming that his SARS-CoV-2 reinfection was likely the result of a broad geographic reintroduction and unlikely to represent an instance of persistent SARS-CoV-2 infection.

Given our findings of the distinct genetic lineages of each SARS-CoV-2 isolate, the lack of multiple strains of SARS-CoV-2 during reinfection, and the congruent geographic patterns of the patient’s clinical narrative, we established that our case represents a genetically confirmed SARS-CoV-2 reinfection and next sought to identify the specific immune correlates that conferred this susceptibility.

### Immunologic profiling reveals naïve lymphocyte depletion and poor humoral immunity

**Figure 3:**
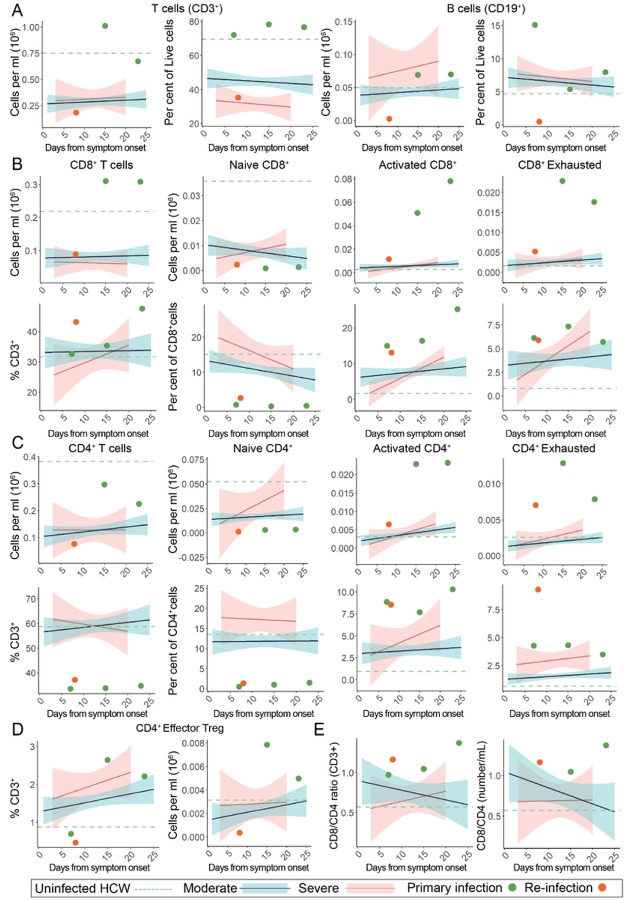
Peripheral lymphocyte profiling of SARS-CoV-2 primary and reinfection demonstrates persistent T cell exhaustion and loss of B cells. For all graphs, blue linear least squares regression lines and corresponding shading represent the average trend and error bars, respectively, for patients with moderate COVID-19. Red linear least squares regression lines and corresponding shading represent the average trend and error bars, respectively, for patients with severe COVID-19. The dashed green line represents the average value of healthy, uninfected healthcare workers (HCW) plotted as a constant value across all days for reference. Individual scatter points represent the values for the patient during the primary SARS-CoV-2 infection (green) at 7, 15, and 23 DFSO and the reinfection (orange) at 8 DFSO*. (A) Total T cells and B cells isolated from patient whole blood. (B) CD8+ T cell subsets plotted as number (top) and relative percentage of parent (bottom). (C) CD4+ T cell subsets plotted as number (top) and relative percentage of parent (bottom). (D) CD4+ TFH cell subsets plotted as number and percentage of parent CD3+. (E) CD8+ / CD4+ ratios calculated relative to days from symptom onset.

**Figure 4:**
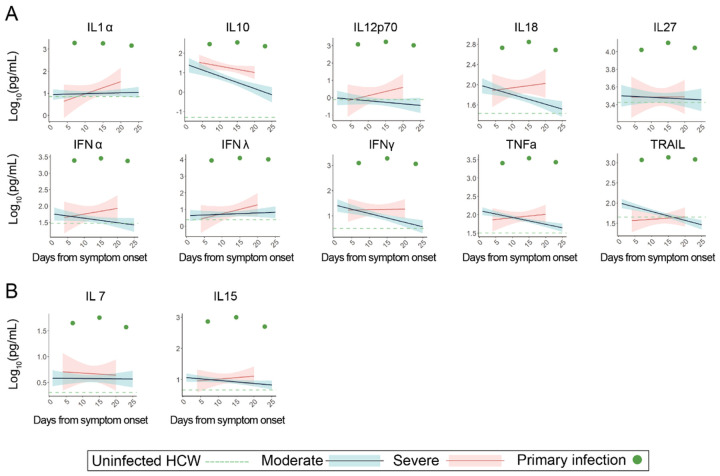
Peripheral cytokine profiling demonstrates broad increases in activation markers suggestive of chronic immune engagement. For all graphs, blue linear least squares regression lines and corresponding shading represent the average trend and error bars, respectively, for patients with moderate COVID-19. Red linear least squares regression lines and corresponding shading represent the average trend and error bars, respectively, for patients with severe COVID-19. The dashed green line represents the average value of healthy, uninfected healthcare workers (HCW) plotted as a constant value across all days for reference. Individual scatter points represent the values for the patient during the primary SARS-CoV-2 infection (green) at 7, 15, and 23 DFSO. (A) Serial measurements of various cytokines plotted against days from symptom onset (B) Select cytokines responsible for naïve T-cell proliferation and maintenance

During the patient’s primary SARS-CoV-2 infection, we performed longitudinal whole blood sampling which was separated into peripheral blood mononuclear cells (PBMC) and serum fractions at 7, 15, and 23 DFSO. PBMCs were analyzed by multidimensional flow cytometry and serum was analyzed with multiplex ELISA to measure 71 cytokines (Fig. 3–4; Extended Data Fig. S3-S4).

In comparison to disease severity and DFSO matched patients from our larger IMPACT cohort, we found that the patient differed significantly in both immune cell subtype composition as well as cytokine expression during his primary infection. Notably, during the primary SARS-CoV-2 infection, the patient maintained very high levels of circulating T-cells and did not suffer from a T-cell lymphopenia as is characteristic of symptomatic COVID-19 patients^32^ (Fig. 3a). Not only was general lymphopenia absent, there also was no specific loss of CD8^+^ T cells, as can be seen in more severe cases of COVID-19^33,34^ (Fig. 3b). Importantly the patient also demonstrated a relatively higher, rather than characteristically depressed, CD8^+^/CD4^+^ ratio primarily as a result of his diminished CD4^+^ populations. (Fig. 3b–c,e; Extended Data Fig S3b). With regards to functionality, the patient’s CD8^+^ and CD4^+^ T-cells exhibited broad increases activation markers (CD38^+^, HLA-DR^+^), exhaustion/terminal differentiation markers (PD1^+^, TIM-3^+^), and effector T regulatory cell markers (PD1^+^, TIM-3^+^, CD25^+^, CD127^−^, HLA-DR^+^) (Fig. 3b–d). In comparison to the larger IMPACT cohort, this patient’s immunological profile was uncharacteristic of either moderate or severe SARS-CoV-2 infection, and instead resembled an immunophenotype consistent with chronic antigen exposure. Importantly, we found that the patient also had very low numbers of circulating naïve CD4^+^ and CD8^+^ T cells at the time of primary SARS-CoV-2 infection, which are required for the generation of potent de novo antiviral response.

To assess whether alterations in immune cell composition contributed to reinfection, we again performed multidimensional flow cytometry on PBMCs isolated from the patient at 5 DFSO* (Fig. 3a–c, orange). In comparison to results from the primary infection (Fig. 3a–c, green), we found a general loss of circulating lymphocytes, while myeloid cell subsets remained at similar levels as seen during his primary infection (Extended Data Fig. 3S). We suspect that this broad depletion of lymphocytes was due to intervening treatment with antithymocyte globulin and rituximab during an episode of graft rejection 3 months prior to his reinfection (Fig. 1). It is also possible that the SARS-CoV-2 reinfection exacerbated this global depletion, although decreases in circulating B cell populations have not been widely reported in COVID-19 patients. Among the patient’s remaining T cell populations, and in the context of recent anti-rejection treatment, the patient again presented with largely depleted pools of naïve CD4^+^ and CD8^+^ T-cells and with continued activation and exhaustion among effector CD4^+^ and CD8^+^ T cell populations. In contrast to the primary infection, we found an almost complete depletion of CD19^+^ B cells, likely as a result of intervening rituximab treatment (Fig. 3a). Consistent with our findings during the primary infection, the patient again presented with an immunophenotype suggestive of chronic antigen engagement, but with globally reduced lymphocytes likely by the treatment for TMCR and AMR.

To investigate the full extent of immunological dysfunction present in the patient during the primary SARS-CoV-2 infection, we next explored whether altered cytokine signaling could have contributed to the patient’s poor initial adaptive immune response. Accordingly, we performed multiplex cytokine analysis from the patient’s serum and found that the patient had globally elevated cytokines (Extended Data Fig. S4) including IL-10, IFN , IFNλ, IL-1 , TNF , TRAIL, and IL-27 at all sampled points during primary infection. Other markers of T-cell functionality, including secreted cytokine IFNγ and T cell activating cytokines IL-18 and IL-12, remained elevated through the patient’s course of infection even after improvement in COVID-19 symptoms (Fig. 4a). In contrast to this patient, a disease-severity matched COVID-19 cohort showed either no elevation, or conversely, a reduction in levels of these cytokines over their course of infection. Additionally, the patient’s IL-15 and IL-7 levels, required for maintenance of naïve T-cell pools, were also persistently elevated (Fig. 4b). These data suggest that persistent utilization of T-cell populations - likely a result of continual immunological response to the patient’s allograft - rather than poor production of cytokines may be responsible for low numbers of naïve T cells at the time of primary SARS-CoV-2 infection.

**Figure 5:**
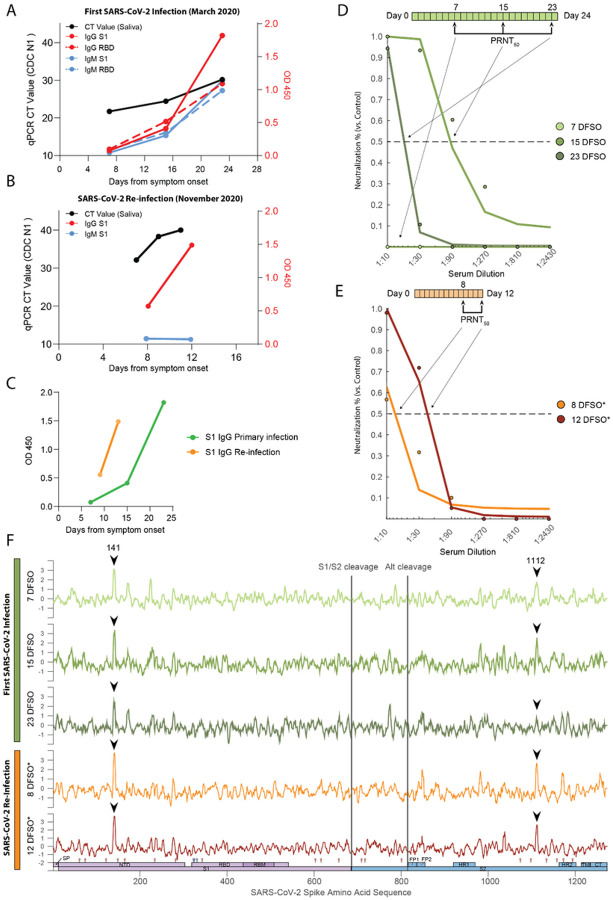
Humoral responses to primary and recurrent SARS-CoV-2 infection. (A) SARS-CoV-2 spike S1 (S1) and receptor binding domain (RBD) ELISA values, measured at optical density 450 (O.D. 450), as observed during the patient’s primary SARS-CoV-2 infection. Values are plotted against days from symptom onset (DFSO). Cycle threshold (CT) RT-qPCR values are shown in black (left y-axis) and corresponding ELISA data is shown in red (IgG) and blue (IgM) (right y-axis, red). Solid lines correspond to S1, dashed lines correspond to RBD (B) S1 ELISA values from the patient’s SARS-CoV-2 reinfection in November of 2020. CT qPCR values are shown in black (left axis) and corresponding ELISA data is shown in red (IgG) and blue (IgM) (right axis). Values are plotted against reinfection days from symptom onset (DFSO*) (C) ELISA S1 IgG trajectories plotted from primary and reinfection (D, E) Longitudinal PRNT50 assays for each sample collected during the patient’s SARS-CoV-2 primary (top, green) and reinfection (bottom, orange) episodes. Dots represent neutralization of the patient’s serum relative to healthy, uninfected healthcare workers. Solid lines represent the best fit of a generalized linear model for estimating serum IC50 values. Condensed clinical timeline (above) shows timing of PRNT50 assays relative to days from symptom onset. (F) PIWAS tiling data representing binding locations of patient’s antibodies against Spike protein. Samples are ordered longitudinally by rows (primary infection (green); reinfection (orange)) to track humoral dynamics. Shared peaks and respective peak heights between the primary and reinfection are annotated (black arrows). A map of SARS-CoV-2 spike domains is provided for reference against antibody binding locations (bottom).

Given the patient’s loss of B cells prior to reinfection following the administration of rituximab (Fig. 1, Fig. 3), we initially hypothesized that SARS-CoV-2 reinfection may have also been the result of loss of humoral immunity. Accordingly, we first assessed anti-SARS-CoV-2 IgG and IgM levels by ELISA during primary infection and found that the patient produced typical levels of SARS-CoV-2 specific antibodies (S1 and RBD) compared with other hospitalized COVID-19 patients (Fig. 5a). Increasing S1 IgG and IgM levels positively correlated with rising RT-qPCR CT values specific for SARS-CoV-2 genomes (i.e. decreasing viral load), suggesting their role in resolution of the primary SARS-CoV-2 infection. During the patient’s reinfection, and in the setting of few circulating B-cells, we found an accelerated S1 IgG response that was again positively correlated with RT-qPCR CT values, suggestive of a memory response upon pathogen rechallenge (Fig. 5b–c). Moreover, there was a complete absence of S1 specific IgM during reinfection, consistent with a memory response to SARS-CoV-2 infection (Fig. 5b). These results suggest that antiviral antibodies were not lost during rituximab treatment as initially hypothesized, and furthermore that the source of S1-specific IgG during the reinfection was likely due to long-lived plasma cells (which are not depleted by rituximab^35^) generated during initial SARS-CoV-2 infection rather than a de novo response to the reinfection.

To assess the neutralizing capacity of anti-SARS-CoV-2 antibodies present during both the primary and recurrent SARS-CoV-2 infections, we performed longitudinal PRNT_50_ assays and calculated the corresponding serum dilution IC_50_ values for each time point (Fig. 5d–e). While the patient developed neutralizing antibodies by 15 DFSO, they were transient in nature and significantly declined in potency by 23 DFSO. This atypical neutralizing antibody response is not consistent with other large-scale studies that show persistence of neutralization capacity following SARS-CoV-2 infection (t_1/2_=90 days; 95% CI: 70–125 days). Furthermore, the neutralization capacity was notably reduced even in comparison to other hospitalized COVID-19 patients of matched disease severity (Extended Data Fig. S5a-b). Longitudinal analysis of serum samples was not performed during the intervening period between primary infection and reinfection; however, early hospital clinical laboratory serologic assays showed persistence of anti-SARS-CoV-2 IgG at 5 DFSO*. We were able to assess neutralizing antibodies during the reinfection, and found that neutralizing antibodies were present at 8 DFSO* and increased slightly by 12 DFSO*. Similar to the primary infection they were of poor neutralizing capacity relative to other COVID-19 patients (Fig S5a; Extended Data Fig. S6). Given that the patient was depleted of naïve circulating B cells, had no IgM response, and had detectable circulating antibodies as early as 5 DFSO*, we hypothesized that these neutralizing antibodies observed during the reinfection reflected antibodies generated from the primary infection, rather than a new humoral response to the reinfection. To examine whether these neutralizing antibodies targeted the same regions with in the SARS-CoV-2 spike protein, we performed linear epitope mapping of this patient’s antibody binding using Serum Epitope Repertoire Analysis (SERA) - a random bacterial display peptide library - coupled with a recently described bioinformatic method that enriches for antigen-specific antibody binding signals relative to healthy (uninfected) controls (Protein-based Immunome Wide Association Study, “PIWAS”)^36^. Using this approach, we found two characteristic PIWAS peaks - signifying locations of peak patient antibody binding - at identical locations in both the primary infection and reinfection (Fig. 5f, black arrows). These peaks of antibody binding were centered on amino acid 141 in the N-terminal domain of S1 and on amino acid 1112 in the S2 domain of Spike. The high degree of concordance in peak locations between primary infection and reinfection suggests the same antibody-secreting population responded to both infections. Importantly, this peak is distinct from the Spike amino acid mutation at 1078 that was found only in the reinfection isolate (Extended Data Fig. S2), suggesting that viral evasion of the antibody response generated during the primary infection was unlikely to be responsible for reinfection.

In summary, we found that the patient developed an antigen-specific, neutralizing antibody response during his primary SARS-CoV-2 infection; that this neutralizing antibody response likely developed into a long-lived plasma cell population; and that it was insufficient to provide protection against reinfections with a novel lineage of SARS-CoV-2 that bore no evidence of viral immune evasion.

## Discussion

We have described a case of symptomatic SARS-CoV-2 reinfection in a solid organ transplant recipient and profiled the unique immunological dysfunctions present during both initial SARS-CoV-2 infection and reinfection. Through extensive clinical investigation and phylogenetic analysis of virus sequences, we confirmed that the patient was reinfected with a genetically distinct lineage of SARS-CoV-2, which was neither the result of persistent infection nor the result of infection by an antigenically distinct SARS-CoV-2 variant. Accordingly, we investigated the potential mechanistic causes of this patient’s multiple SARS-CoV-2 infections by performing longitudinal immunologic profiling during both initial SARS-CoV-2 infection and reinfection.

Multiple recent longitudinal studies have shown that the majority of COVID-19 patients, even those with mild or asymptomatic infection, develop long-lasting SARS-CoV-2 specific cellular and humoral adaptive immunity for as long as 8 months^37^. In contrast, a series of case reports and this manuscript report that SARS-CoV-2 reinfections can occur between 48 and 236 days from initial infection (Extended Data Table 1). The discrepancy between frequent, durable protective immune responses generated during most SARS-CoV-2 infections and rare cases of reported reinfection by antigenically similar variants is currently unexplained. While the protective capacity of humoral responses against SARS-CoV-2 infection is apparent, large variability in magnitude of responses between patients has been shown in multiple longitudinal studies. The underlying immunologic correlates of this variability, and ultimately what is required to develop strong humoral responses, have not been fully elucidated.

In this case study, we found that a failure of humoral immunity may have led to this patient’s SARS-CoV-2 reinfection. We investigated both the dynamics of antibody production as well as the general quality of antibodies produced by the patient. Our initial analysis of humoral responses indicated that the patient mounted a typical IgM and IgG response to SARS-CoV-2 primary infection, as assessed by longitudinal ELISA measurements. While total anti-SARS-CoV-2 antibody production was not particularly hampered in this patient, the neutralizing antibody response was clearly defective.

To address the underlying cellular defects that may have led to a poor neutralizing antibody response, we performed flow cytometry analysis of PBMC populations during primary infection and reinfection. We found significant differences in the patient’s T cell composition and phenotype relative to other patients with COVID-19. While T cell lymphopenia is common in even mild cases of COVID-19 and a characteristic response to many other viral infections, this patient did not develop T cell lymphopenia, and but instead presented with a profound and relatively specific reduction in naïve T cell pools, most significantly in their CD4 compartment. Reduced naive T cell pools are a characteristic feature of aging and may contribute to the impaired immune responses observed in elderly individuals^38^. Depletions in naive T cell populations, and the corresponding deficits in adaptive immune responses, have also been reported in inflammatory states like chronic hepatitis C infection^39^ and chronic granulomatous disease^40^; however, this phenomenon has been less well documented in solid organ transplantation. Additionally, it has been consistently observed that solid organ transplant recipients develop poor adaptive response to new antigens either during immunization or new infections - including SARS-CoV-2 mRNA vaccination^41–43^. It is unlikely that the patient’s immunosuppression prior to primary infection led to naïve T cell specific lymphopenia as MMF, a purine biosynthesis inhibitor, would be expected to inhibit both T and B cell proliferation non-specifically, and this patient, having received Belatacept, a CTLA-4 F_c_ fusion protein and co-stimulatory inhibitor, would also be expected to inhibit T cell activation and differentiation, which would more likely lead to increased, rather than decreased, naive T cell pools. Additionally, cytokine profiling revealed high levels of IL-7 and IL-15, both of which promote naive T cell pool expansion^44,45^. While it is clear that the patient had sufficient cytokines to replenish naive T cell pools, naive T cell populations were not replenished, possibly due to either over-utilization (via repeated antigen engagement), insufficient thymic reserve, or some combination of both. Interestingly, immunophenotyping also revealed high levels of activation, terminal differentiation, and exhaustion in both CD4^+^ and CD8^+^ pools, possibly as a result of chronic antigen exposure from the transplanted organ^46^. We suspect that the lack of naive T cell pools may have contributed to a deficient humoral immune response during initial SARS-CoV-2 infection. Whether similar impaired cellular dynamics may lead to impaired humoral immunity to SARS-CoV-2 in other long-term organ graft recipients, or other populations with aspects of repeated antigen exposure such as chronic infection and cancer, warrants further investigation.

While the patient’s overall anti-SARS-CoV-2 response was not specifically impaired by low naïve CD4 T cell pools, we suspect that the patient’s poor neutralization response may have been and that the insufficient T cell support resulted in either extrafollicular or dysfunctional germinal center B cell responses^34,47–49^. In line with these findings, the patient demonstrated a transient, relatively poor-quality neutralizing antibody response during initial infection consistent with a short-lived extrafollicular response or deficient germinal center dynamics.

SARS-CoV-2 specific IgG antibodies were detected as early as 5 DFSO* during reinfection, suggesting a memory response given the accelerated humoral kinetics relative to first infection. Additionally, the absence of circulating B cell and antiviral IgM during the reinfection indicates that it was likely only SARS-CoV-2 specific plasma cells established after the primary infection that provided neutralizing antibodies during reinfection. To better characterize these neutralizing antibodies, we performed linear epitope profiling of SARS-CoV-2 specific antibodies from the primary infection and reinfection against the spike protein. This revealed binding peaks at identical amino acid locations, suggesting that a humoral memory response was indeed generated during the patient’s primary infection and was also present during reinfection. Importantly, the spike protein amino acid change we identified in the virus strain causing the SARS-CoV-2 reinfection did not correspond to the location of antibody binding generated during the primary infection, suggesting viral evasion of primary humoral responses to be an unlikely explanation for reinfection in this case (Fig. 5f, Extended Data Fig. S2). These two binding sites within the spike protein corresponded to amino acid 141 and 1112, which did not correspond to areas of high antigenicity or neutralization with in our larger IMPACT cohort (Extended Data Fig. S6), We hypothesize that the patient’s underlying immune deficiencies (low naïve CD4 pools) led to poor neutralizing antibody quality (IC_50_ titers approximately 1:10 to 1:30), which were insufficient to protect against SARS-CoV-2 reinfection *in vivo*.

By means of a case study of a solid organ transplant recipient with COVID-19, we demonstrate that the mere presence of neutralizing antibodies during primary infection was insufficient to confer protection against reinfection. Further investigation into additional immunological correlates of protection, including the roles of cellular immunity and tissue-resident immune cell populations, are warranted.

## Limitations Of The Study

As with all case studies, a limitation on the generalizability of our findings to wider patient populations is present. Also, while the lack of immunological responsiveness to vaccination or acute infection in immunosuppressed and solid organ transplant populations is well documented, there may be additional mechanisms contributing to these defects beyond those discussed in this manuscript - particularly with regards to SARS-CoV-2 infection. Our analysis of the immunophenotype of the patient was limited to surveys of circulating immune dynamics; however, numerous studies have also described perturbations in immunity at tissue sites not easily amenable to direct interrogation. We also did not directly analyze antigen specific T-cell responses during either infection, which may reveal additional dysfunction not discussed within this manuscript. Lastly, we also did not fully address every potential avenue of viral immune evasion to immune responses and accordingly suggest that a greater understanding of the virus-intrinsic and host-intrinsic features determining susceptibility to SARS-CoV-2 reinfections is required. Future studies should investigate not only the circulating and systemic adaptive immune responses during SARS-CoV-2 reinfections, but also the possibility that local defects in immune responsiveness among barrier tissue sites may also enable recurrent SARS-CoV-2 infection.

## Supplementary Material

Supplement 1
