# Supplementary material for: Longitudinal immune profiling of a SARS-CoV-2 reinfection in a solid organ transplant recipient
Source: Res Sq. 2021 May 5:rs.3.rs-405958. Preprint. [Version 1] doi: 10.21203/rs.3.rs-405958/v1 (PMC8132249; doi:10.21203/rs.3.rs-405958/v1)
Supplement: Supplement 1 [file d09011390b0dfabce1be4697.docx]

**Extended Data**

**Extended Data Table S1**


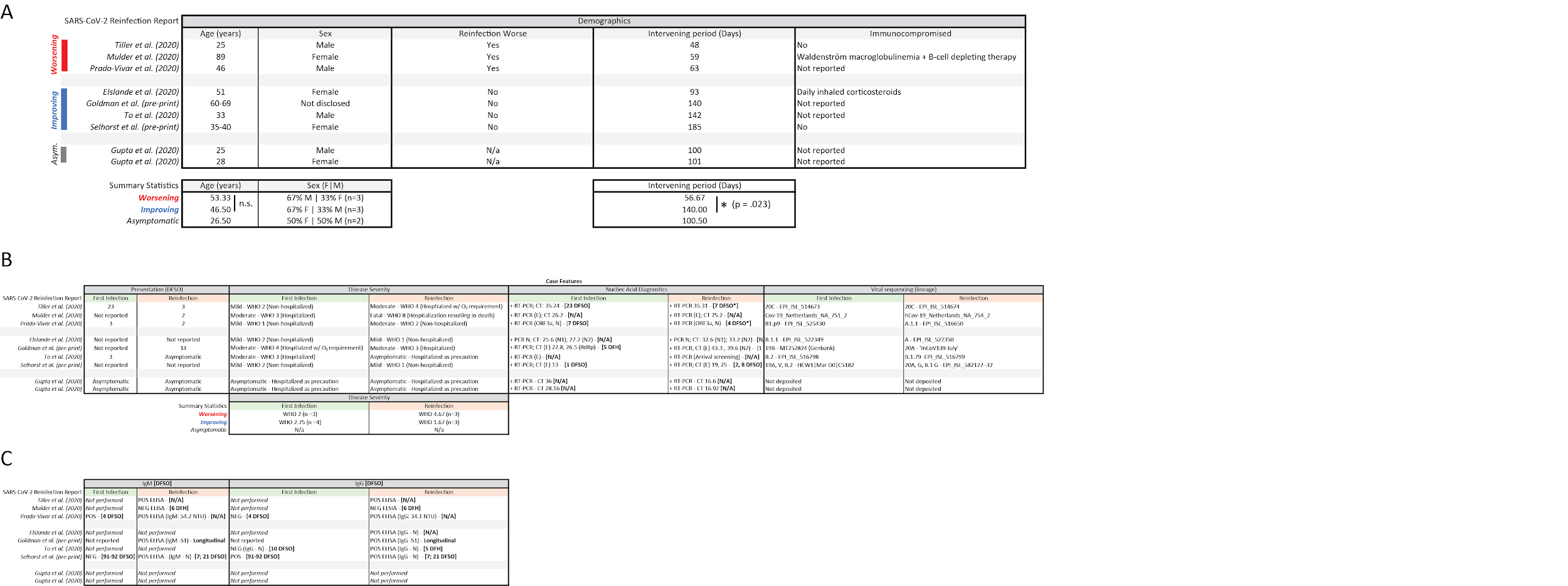


**Extended Data Table 1. Literature summary of SARS-CoV-2 reinfection reports.** **(A)** Demographic and clinical features of SARS-CoV-2 reinfection reports, stratified by clinical progression (worsening, improving, asymptomatic) and ordered by length of intervening period between infections (shortest first). Summary statistics are grouped below in corresponding columns. Group averages between ‘Worsening’ and ‘Improving’ were compared for significance using a two-samples t-test, with equal variances between groups for ‘Age’, and unequal variances for ‘Intervening Period’ as determined by results from F-test statistics. **(B)** Corresponding case features for SARS-CoV-2 reinfection reports stratified by primary infection (green) and reinfection (orange). DFSO represents reported days from symptom onset for primary (green) and reinfection (orange) cases. World Health Organization (WHO) COVID-19 disease severity was assigned retrospectively during the course of this review and were not supplied by the original authors. Where available, nucleic acid results are reported as: Type of test (nucleic acid target): CT values [DFSO or days from hospitalization (DFH)]. Accession numbers for viral genomes are reported as provided in manuscripts. **(C)** Serological results for SARS-CoV-2 reinfection reports stratified by primary infection (green) and reinfection (orange). IgM and IgG data are presented as reported in manuscripts according to the following scheme: Type of test / Result of Test: Quantitative value [DFSO or DFH].

**Extended Data Table S2**

| **DFSO** | **DSFO*** | **Nasopharyngeal swab** | | | | **Saliva sample** | | | |
| --- | --- | --- | --- | --- | --- | --- | --- | --- | --- |
|  |  | **N1** | **N2** | **RP** | **Result** | **N1** | **N2** | **RP** | **Result** |
| 3 |  | 17.1 | 16.3 |  | POS |  |  |  |  |
| 7 |  | ND | ND | 30.39 | NEG | 21.7 | 22.6 | 21.5 | POS |
| ^#^10 |  |  | 18.7 |  | POS |  |  |  |  |
| 11 |  | 19.6 | 20.3 | 26.3 | POS |  |  |  |  |
| 13 |  | 21.6 | 21.2 |  | POS |  |  |  |  |
| 15 |  | 29.7 | 30.9 | 24.6 | POS | 24.4 | 26.0 | 21.4 | POS |
| 16 |  | 31.5 | 31.2 |  | POS |  |  |  |  |
| 19 |  | 33.2 | 34.5 | 23.0 | POS |  |  |  |  |
| 19 |  | 29.5 | 30.8 |  | POS |  |  |  |  |
| 22 |  | 25.7 | 26.2 |  | POS |  |  |  |  |
| 23 |  | 39.1 | 41.3 | 28.6 | INC | 30.2 | 33.0 | 18.5 | POS |
| 27 |  | 30.2 | 31.0 |  | POS |  |  |  |  |
| ^$^109 |  |  |  |  | NEG |  |  |  |  |
| ^$^220 |  |  |  |  | NEG |  |  |  |  |
| ^$^236 | 2 |  |  |  | NEG |  |  |  |  |
| 237 | 3 | 27.34 | 27.15 |  | POS |  |  |  |  |
| 239 | 5 | 34.2 | 34.9 | 27.6 | POS | 32.1 | 32.7 | 27.1 | POS |
| 241 | 7 | 25.4 | 25.3 | 26.3 | POS | 38.3 | ND | 24.8 | NEG |
| ^$^242 | 8 |  |  |  | POS |  |  |  |  |
| 243 | 9 | 36.0 | 36.1 | 32.3 | POS | ND | ND | 23.4 | NEG |
| 245 | 11 | 31.7 | 31.8 | 31.9 | POS | ND | ND | 24.8 | NEG |

**Extended Data Table 2: RT-qPCR results for nasopharyngeal swabs and saliva specimens**. DFSO corresponds to days from symptom onset during the patient’s primary SARS-CoV-2 infection, whereas DFSO* corresponds to days from symptom onset relative to the SARS-CoV-2 reinfection. Results of nucleic acid testing from each day and for each sample type are reported in consecutive rows ordered by DFSO. N1 and N2 columns correspond to CDC-N1 and CDC-N2 primer sets for the detection of SARS-CoV-2 nucleic acids. RT-qPCR cycle threshold (CT) values are reported for various samples, with “ND” indicating SARS-CoV-2 nucleic acids were not detected. As previously described^50^, summary test results are stratified by CDC-N1 CT values according to the following scheme: positive (“POS”) = CT ≤ 38, negative (“NEG”) = CT ≥ 40, or Inconclusive (“INC”) = CT ≥ 38 and CT ≤ 40. Black text indicates samples processed by research lab. Red text indicates samples processed by clinical lab. ND= not detected. # run on Cepheid platform. $ run on Panther platform.

**Extended Data Figure S1**

**
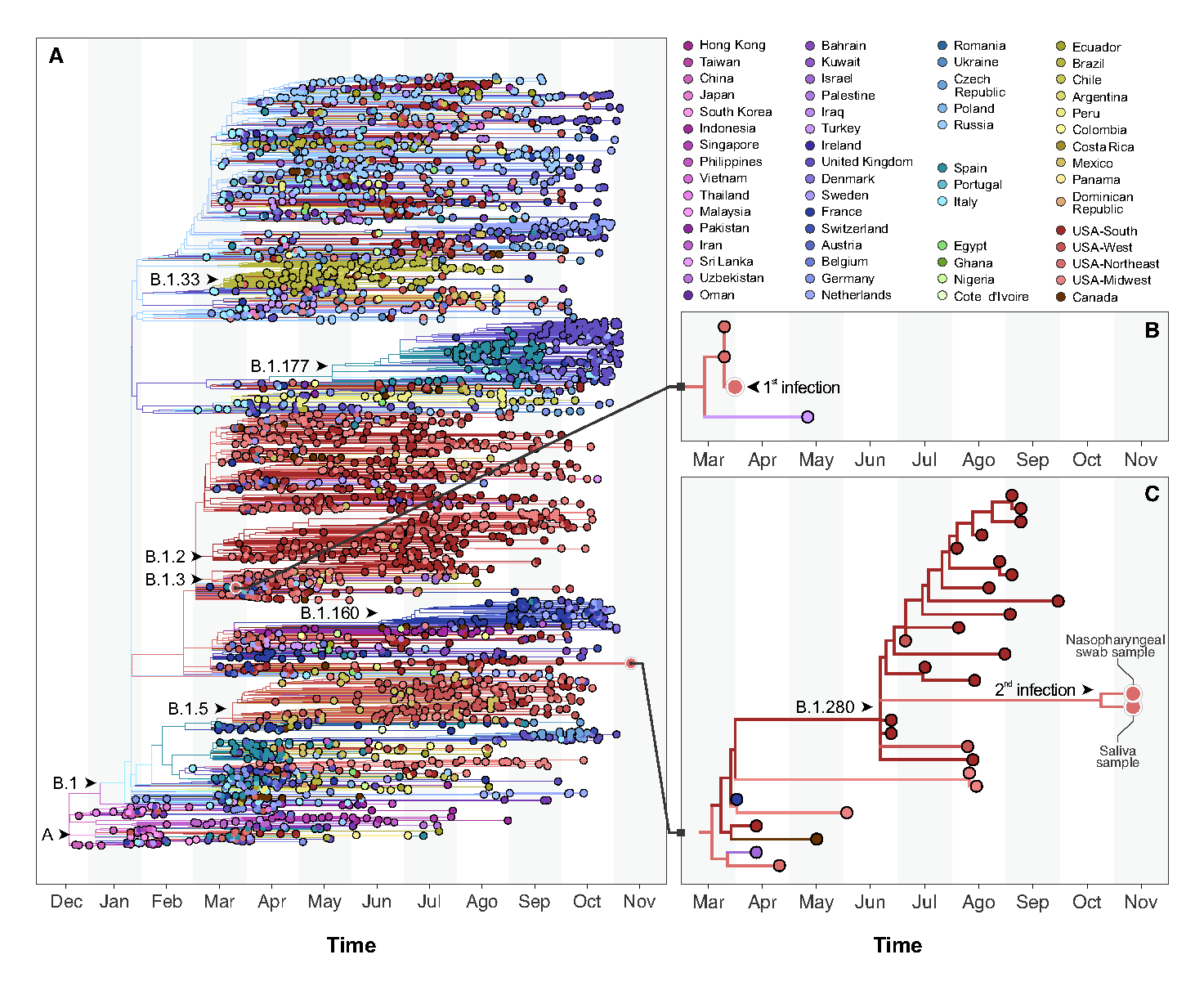
**

**Extended Data Figure S1.** **Large phylogeny of SARS-CoV-2 whole genomes**. **(A)** Maximum likelihood tree reconstructed with 3,068 global viral genomes from GISAID, sampled to represent the diversity of viruses in circulation since the beginning of the COVID-19 pandemic. This phylogeny revealed the placement of the viruses infecting the patient in distinct lineages/sub-lineages of SARS-CoV-2. **(B)** The virus causing the primary infection was isolated in March 2020 and belongs to lineage B.1. **(C)** The virus causing the reinfection was collected in November 2020 and is found in the sublineages B.1.208. Phylogenetic plots were done using baltic 0.1.0.

**Extended Data Figure S2**

**
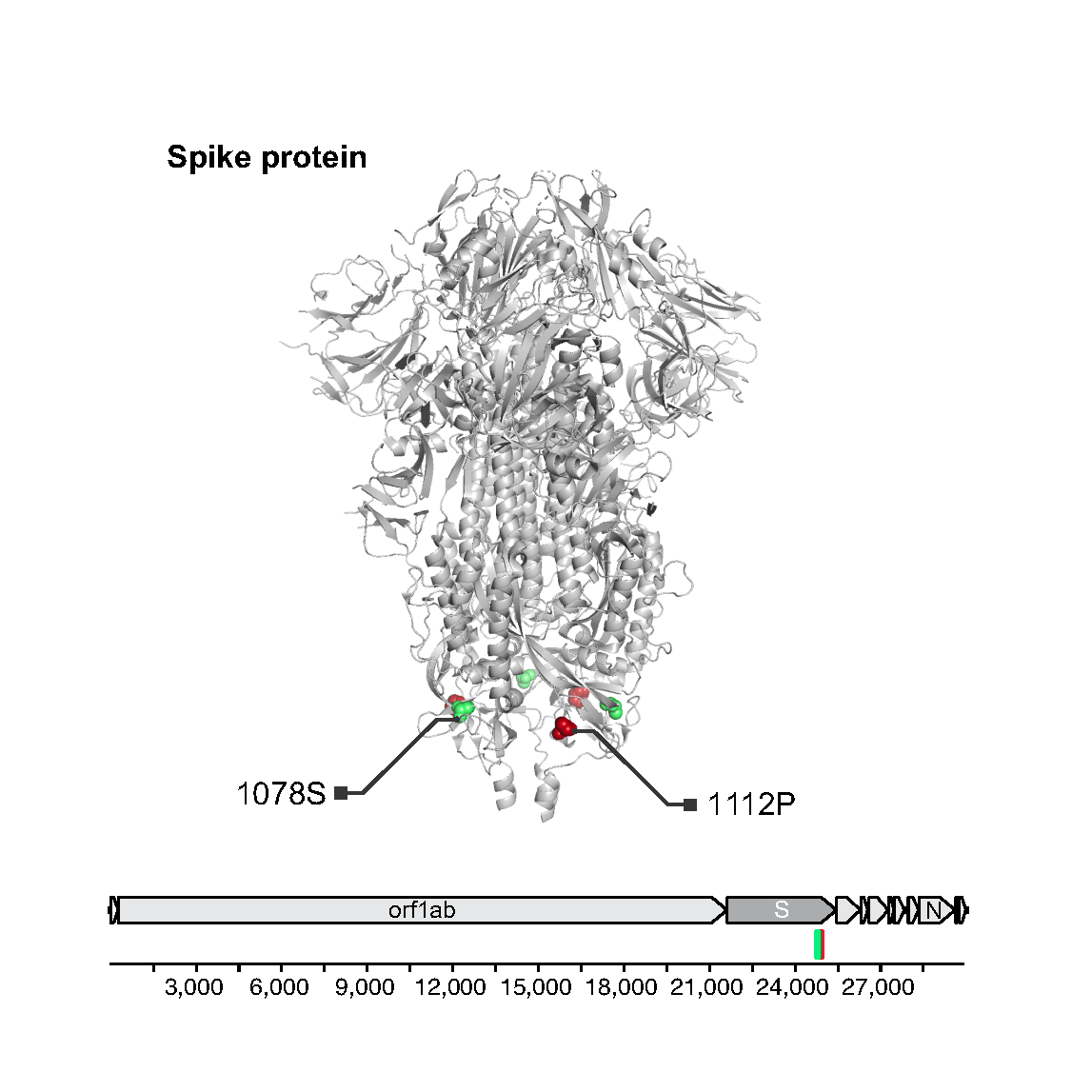
Extended Data Figure S2. A non-synonymous amino acid change in the genome of the SARS-CoV-2 reinfection variant**. An amino acid change from alanine to serine (A1078S) was discovered in the S2 subunit of spike protein in the SARS-CoV-2 variant that caused the reinfection (green marker). Antibody binding site identified by linear epitope mapping that was generated during primary infection (red). Protein structure: PDB 6VXX^51^

**Extended Data Figure S3**

**
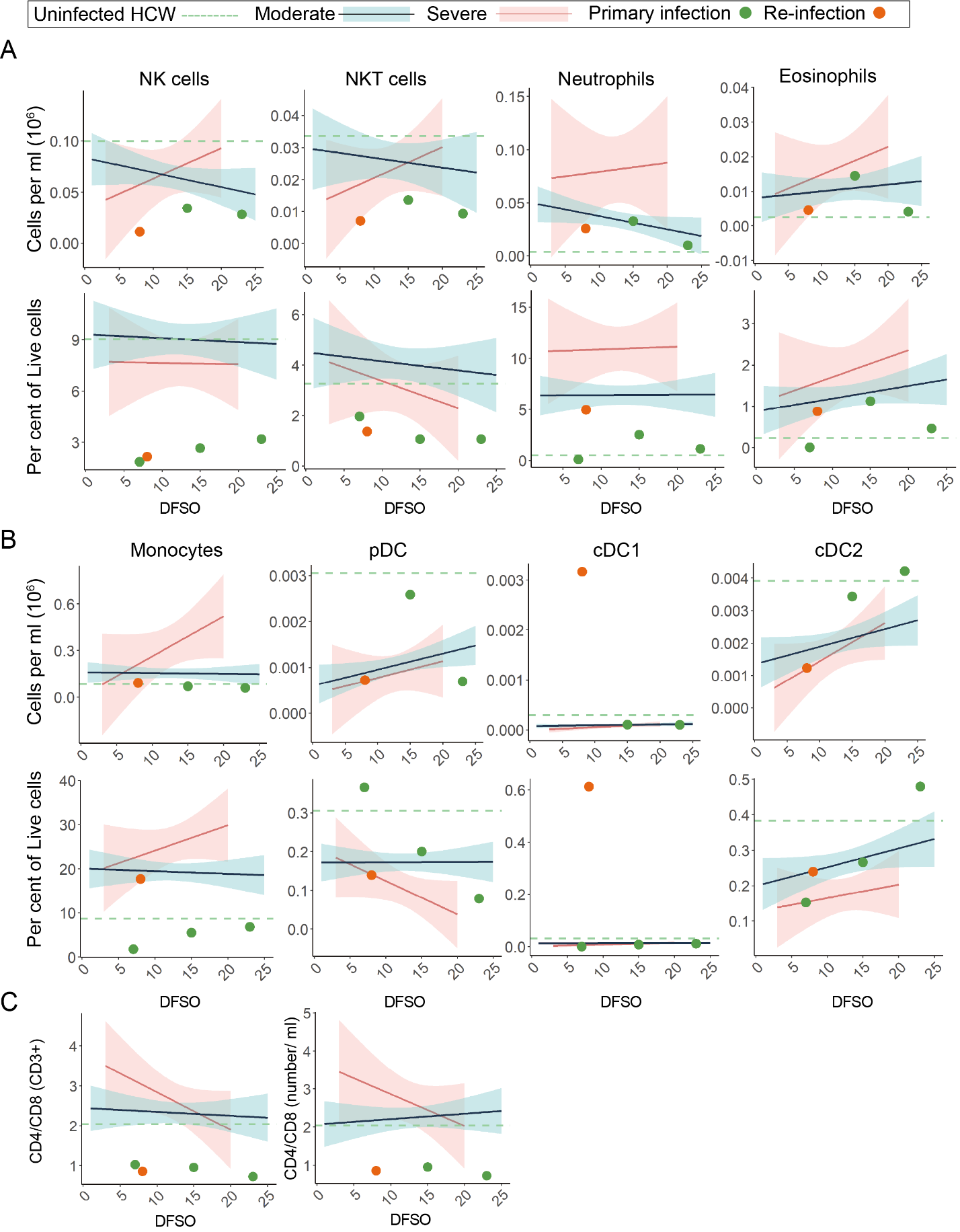
**

**Extended Data Figure S3**. **Broad PBMC profiling of SARS-CoV-2 primary and reinfection**. For all graphs, blue linear least squares regression lines and corresponding shading represent the average trend and error bars, respectively, for patients with moderate COVID-19. Red linear least squares regression lines and corresponding shading represent the average trend and error bars, respectively, for patients with severe COVID-19. The dashed green line represents the average value of healthy, uninfected healthcare workers (HCW) plotted as a constant value across all days for reference. Individual scatter points represent the values for the patient during the primary SARS-CoV-2 infection (green) and the reinfection (orange). **(A)** Scatter plots of various patient peripheral NK and granulocyte cell populations isolated from whole blood. Top rows are absolute counts, bottom rows are percentage of relative parent populations. **(B)** Scatter plots of various patient peripheral monocyte and dendritic cell (DC) sub-populations isolated from whole blood. Top rows are absolute counts, bottom rows are percentage of relative parent populations. **(C)** CD4^+^/CD8^+^ ratios plotted as percentage of CD3^+^ PMBC cells (left) or as a ratio of absolute counts (right).

**Extended Data Figure S4**


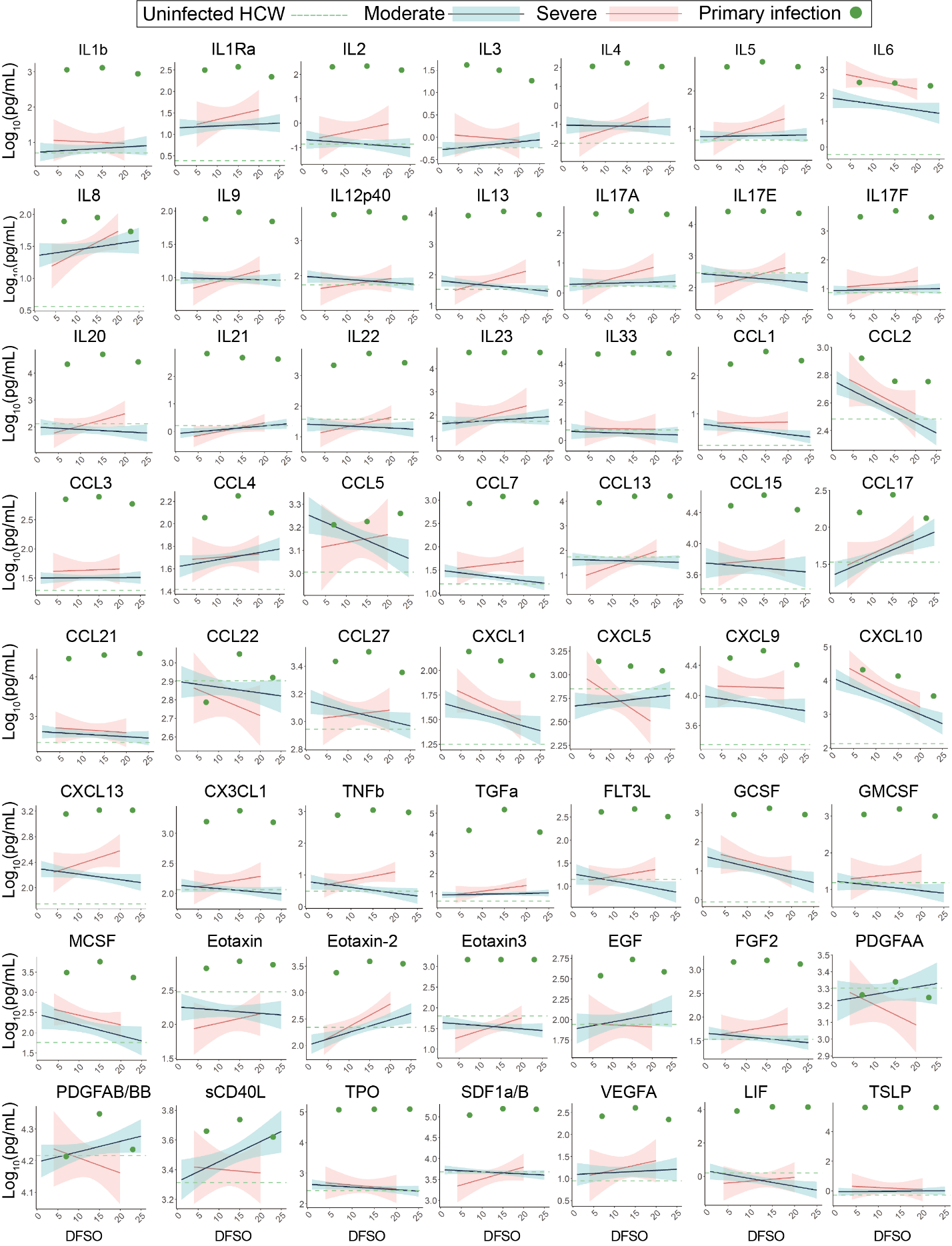


**Extended Data Figure S4**. **Broad plasma cytokine profiling of SARS-CoV-2 primary infection**. For all graphs, blue linear least squares regression lines and corresponding shading represent the average trend and error bars, respectively, for patients with moderate COVID-19. Red linear least squares regression lines and corresponding shading represent the average trend and error bars, respectively, for patients with severe COVID-19. The dashed green line represents the average value of healthy, uninfected healthcare workers (HCW) plotted as a constant value across all days for reference. Individual scatter points represent the values for our patient during the primary SARS-CoV-2 infection (green).

**Extended Data Figure S5**


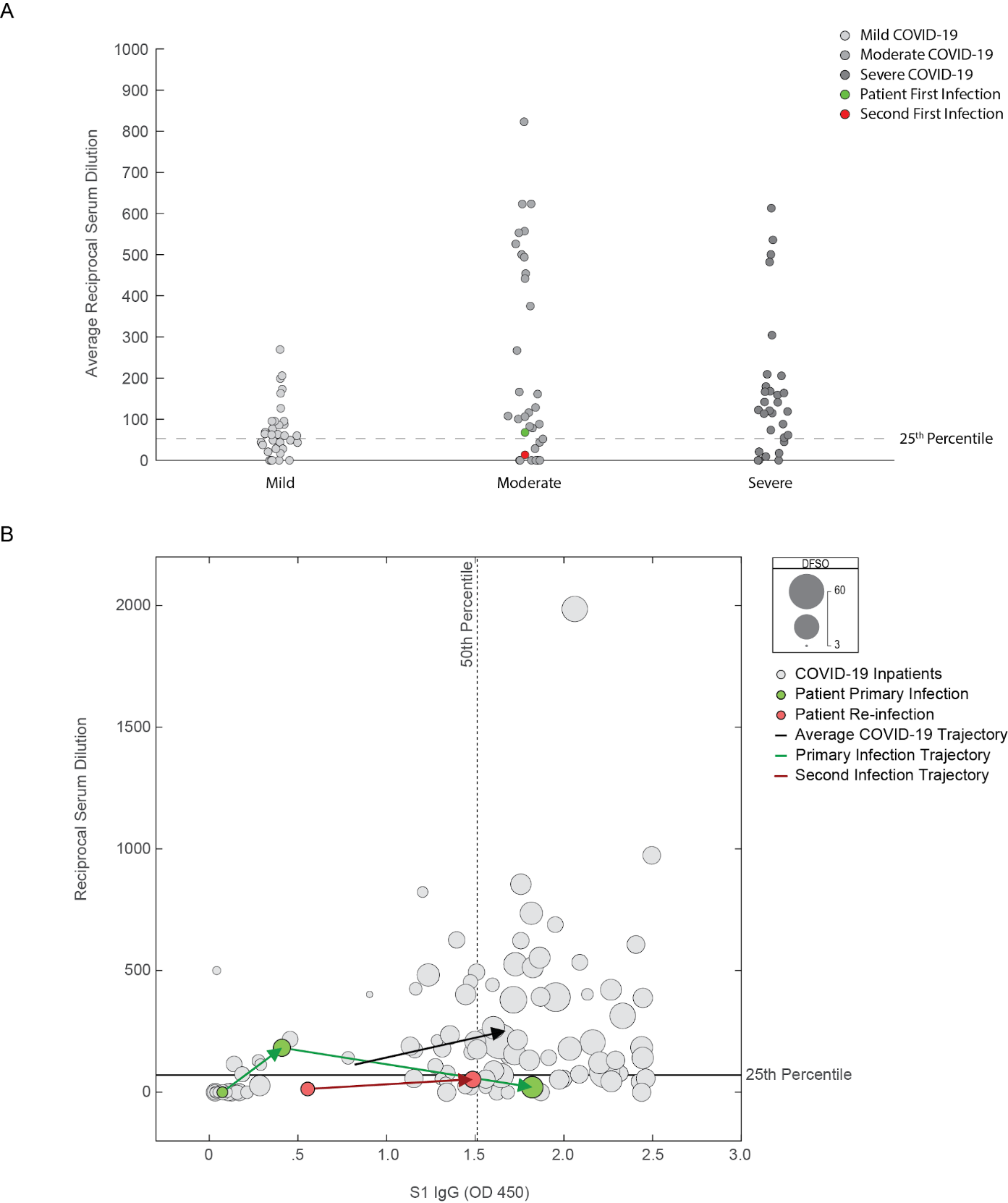


**Extended Data Figure S5**. **Patient’s suboptimal humoral responses as compared to a cohort of COVID-19 patients**. **(A)** Primary infection (green) and reinfection (orange) reciprocal IC_50_ serum dilutions plotted against control COVID-19 patients and stratified by COVID-19 disease severity. Grey points indicate the average reciprocal serum dilution for an individual patient. Dashed line represents the 25^th^ percentile value among patients with COVID-19 who mounted a neutralizing antibody response. **(B)** Reciprocal serum dilution values plotted against corresponding S1 IgG values for each sample collected. Scatter size is scaled against days from symptom onset for each patient. Dashed line indicates the 50^th^ percentile value of IgG response, whereas the solid line represents the 25^th^ percentile reciprocal serum dilution IC_50_ value among patients that mounted a neutralizing antibody response. Green arrows indicate the temporal progression from sequential samples during the primary infection. Orange arrows indicate the temporal progression from sequential samples during the reinfection. Black arrow represents the average trajectory of humoral responses for a control COVID-19 cohort.

**Extended Data Figure S6**


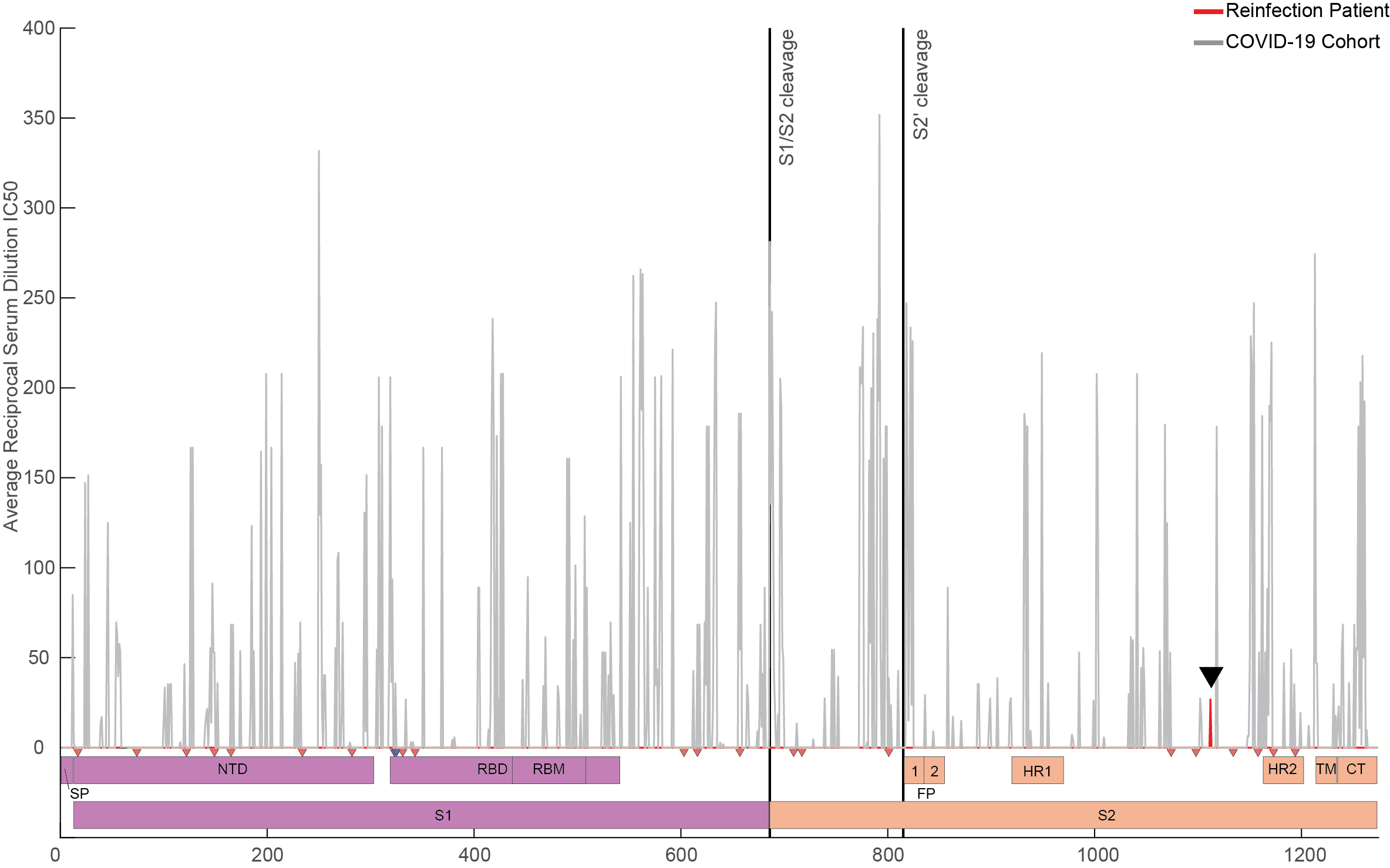


**Extended Data Figure S6**. **Neutralizing antibody landscape of the SARS-CoV-2 spike protein**. Reciprocal serum dilution IC_50_ values are plotted according to identified PIWAS peak locations for hospitalized patients with COVID-19 in the IMPACT cohort (grey). All reciprocal serum dilution values at a given residue were averaged to generate a cohort average value per residue of spike protein. Red peak (black arrow at residue 1112) represents the averaged reciprocal serum dilution IC_50_ for the patient’s primary and reinfections (Peak at 140 not shown). **(Bottom)** Domain map of SARS-CoV-2 spike protein showing S1 (purple) and S2 (orange) regions. N-Glycosylation sites are marked by red triangles, O-linked glycosylation sites are marked by blue triangles. Cleavage sites are marked by solid black lines (“S1/S1 Cleavage” and “S2’ cleavage”).

**Supplementary Information**

**Supplementary Table S1**. **List of genomes used in the analyses**. We gratefully acknowledge the following Authors from the Originating laboratories responsible for obtaining the specimens, as well as the submitting laboratories where the genome data were generated and shared via GISAID, on which this research is based.

**Supplementary Table S2. Yale IMPACT Research Team**

Abeer Obaid^11^, Alice Lu-Culligan^1^, Allison Nelson^10^, Angela Nunez^10^, Anjelica Martin^1^, Anne E. Watkins^2^, Bertie Geng^10^, Christina A. Harden^2^, Codruta Todeasa^10^, Cole Jensen^2^, Daniel Kim^1^, David McDonald^10^, Denise Shepard^10^, Edward Courchaine^11^, Elizabeth B. White^2^, Eric Song^1^, Erin Silva^10^, Eriko Kudo^1^, Giuseppe DeIuliis^10^, Harold Rahming^10^, Hong-Jai Park^10^, Irene Matos^10^, Isabel Ott^2^, Jessica Nouws^10^, Jordan Valdez^10^, Joseph Lim^12^, Kadi-Ann Rose^10^, Kelly Anastasio^13^, Kristina Brower^2^, Laura Glick^10^, Lokesh Sharma^10^, Lorenzo Sewanan^10^, Lynda Knaggs^10^, Maksym Minasyan^10^, Maria Batsu^10^, Maxine Kuang^2^, Maura Nakahata^10^, Melissa Campbell^6^, Melissa Linehan^1^, Michael H. Askenase^14^, Michael Simonov^10^, Mikhail Smolgovsky^10^, Nicole Sonnert^1^, Nida Naushad^10^, Pavithra Vijayakumar^10^, Rick Martinello^3^, Rupak Datta^3^, Ryan Handoko^10^, Santos Bermejo^10^, Sarah Prophet^15^, Sean Bickerton^11^, Sofia Velazquez^14^,Tyler Rice^1^, William Khoury-Hanold^1^, Xiaohua Peng^10^, Yexin Yang^1^, Yiyun Cao^1^, Yvette Strong^10^, Alexander James Robertson^2^, Alice Zhao^2^, Zitong Lin^2^, Coriann E. Dorgay^2^, Natasha C. Balkcom^2^, Caitlin J. Chun^2^

^10^Yale School of Medicine, New Haven, CT, USA.

^11^Department of Biochemistry and of Molecular Biology, Yale University School of Medicine, New Haven, CT, USA.

^12^Yale Viral Hepatitis Program; Yale University School of Medicine, New Haven, CT, USA.

^13^Yale Center for Clinical Investigation, Yale University School of Medicine, New Haven, CT, USA.

^14^Department of Neurology, Yale University School of Medicine, New Haven, CT, USA.

^15^Department of Molecular, Cellular and Developmental Biology, Yale University School of Medicine, New Haven, CT, USA.

**STAR METHODS Legend**

**KEY RESOURCES TABLE**

**RESOURCE AVAILABILITY**

Lead Contacts

Materials Availability

Data Availability

**EXPERIMENTAL MODEL AND SUBJECT DETAILS**

Ethics statement

Patient Case Report and IMPACT Patient Cohort

Cell lines

SARS-CoV-2 Virus

**METHOD DETAILS**

Isolation of patient plasma and PBMCs

SARS-CoV-2 RT-qPCR detection and sequencing

Phylogenetic and molecular evolution analysis

Flow cytometry

SARS-CoV-2 specific-antibody measurements

Enzyme-Linked Immunosorbent Assay (ELISA)

Cytokine and chemokine measurements

Viral neutralization assay

STAR METHODS Text

**RESOURCE AVAILABILITY**

*Lead Contacts*

Correspondence and requests regarding clinical features of this manuscript should be addressed to M.A. Other correspondence and requests should be addressed to A.I.

*Materials availability*

Requests materials should be addressed to B.I. and A.I.

*Data availability*

Requests for data or should be addressed to B.I. and A.I.

**EXPERIMENTAL MODEL AND SUBJECT DETAILS**

*Ethics statement*

This study was approved by Yale Human Research Protection Program Institutional Review Boards (FWA00002571, protocol ID 2000027690). Informed consent was obtained from all enrolled patients and healthcare workers. All work requiring biosafety level three conditions was performed by certified personnel under the supervision of the Yale Department of Environmental Health and Safety.

*Patient Case Report and IMPACT Patient Cohort*

Our case report of a SARS-CoV-2 reinfection was enrolled as part of the broader Yale IMPACT cohort described at length previously and summarized again below for convenience^52,53^. The individual clinical narrative for our SARS-CoV-2 reinfection case was written by two infectious disease fellows and an infectious disease physician specializing in management of solid organ transplant recipients through direct interview of the patient and review of their EMR. All explicit dates in our clinical narrative were converted to relative days from symptom onset as reported by our patient. With regards to the larger IMPACT cohort, and reproduced here for convenience, one hundred and seventy-nine patients admitted to YNHH with COVID-19 between 18 March 2020 and 27 May 2020 were included in this study. No statistical methods were used to predetermine sample size. Patients were scored for COVID-19 disease severity through review of the electronic health records (EMR) at time of each sample collection. Scores were assigned by a clinical infectious disease physician according to a custom-developed disease severity scale. Moderate disease status (clinical scores 1–3) was defined as: (1) SARS-CoV-2 infection requiring hospitalization without supplementary oxygen; (2) infection requiring non-invasive supplementary oxygen (<3 l/min to maintain SpO_2_ >92%); and (3) infection requiring non-invasive supplementary oxygen (>3 l/min to maintain SpO_2_ >92%), or alternatively >2 l/min to maintain SpO_2_ >92% with a high-sensitivity C-reactive protein (hsCRP) >70 and administration of tocilizumab. Severe disease status (clinical score 4 or 5) was defined as: (4) infection meeting all criteria for clinical score 3 and also requiring admission to the ICU and >6 l/min supplementary oxygen to maintain SpO_2_ >92%, or (5) infection requiring invasive mechanical ventilation or extracorporeal membrane oxygenation (ECMO) in addition to glucocorticoid or vasopressor administration. Clinical score 6 was assigned for deceased patients. For all patients, days from symptom onset were estimated as follows: (1) highest priority was given to explicit onset dates provided by patients; (2) next highest priority was given to the earliest reported symptom by a patient; and (3) in the absence of direct information regarding symptom onset, we estimated a date through manual assessment of the EHR by an independent clinician. Symptom onset and etiology were recorded through standardized interviews with patients or patient surrogates upon enrollment in our study, or alternatively through manual EHR review if no interview was possible. Clinical data were collected using EPIC EHR, and de-identified and aggregated using REDCap 9.3.6 software.

**METHOD DETAILS**

*Isolation of patient plasma and PBMCs*

As reported previously and reproduced here for convenience^52,53^, patient whole blood was collected in sodium heparin-coated vacutainers and kept gently agitating at room temperature until sample pick-up by IMPACT team members. All blood was processed on the day of collection. Plasma samples were collected after centrifugation of whole blood at 400*g* for 10 minutes at room temperature (RT) without brake. The undiluted serum was then transferred to 15-ml polypropylene conical tubes, and aliquoted and stored at −80 °C for subsequent analysis. PBMCs were isolated using Histopaque (Sigma-Aldrich, #10771-500ML) density gradient centrifugation in a biosafety level 2+ facility. After isolation of undiluted serum, blood was diluted 1:1 in room temperature PBS, layered over Histopaque in a SepMate tube (StemCell Technologies; #85460) and centrifuged for 10 minutes at 1,200*g*. The PBMC layer was isolated according to the manufacturer’s instructions. Cells were washed twice with PBS before counting. Pelleted cells were briefly treated with an ACK lysis buffer for 2 minutes and then counted. Percentage viability was estimated using standard Trypan blue staining and an automated cell counter (Thermo-Fisher, #AMQAX1000).

*SARS-CoV-2 RT-qPCR detection and sequencing*

Total nucleic acid was extracted from nasopharyngeal swabs and saliva specimens with the MagMAX Viral/Pathogen Nucleic Acid Isolation kit^54^. We used the modified CDC RT-qPCR assay to detect SARS-CoV-2 in the extracted nucleic acid^50,55^. A selection of specimens from both time periods that tested positive were sequenced using a highly multiplexed amplicon sequencing approach as described in the ARTIC Network nCoV-2019 Oxford Nanopore sequencing protocol [(nCoV-2019 sequencing protocol v3 (LoCost) V.3)^56^](https://paperpile.com/c/wuyqKp/B9ski+ptWhQ). Sequencing data were then processed according to the ARTIC bioinformatic pipeline. Briefly, raw .fast5 data was basecalled with Guppy V.4.4.0 and aligned to the reference SARS-CoV-2 genome (Accession MN908947) using minimap2. Primer sequences are masked prior to consensus sequence generation. A threshold of 20X depth of coverage is required to call variants and generate a consensus sequence. Positions in the genome with less than 20X depth of coverage are represented with “N”.

*Phylogenetic and molecular evolution analysis*

To reveal the evolutionary relationship between the virus infecting the patient in March 2020 with those sampled during the reinfection in November 2020, phylogenetic analyses were performed with additional sequences submitted to GISAID (gisaid.org), sampled from all over the world from January to mid-November 2020. Metadata on viral collection time, location, and lineage assignment were also obtained from GISAID. A time series of daily COVID-19 cases per country, obtained from the Center for Systems Science and Engineering (CSSE) data repository at Johns Hopkins University^57^, was used to weight the genome subsampling according to the daily case counts. This allowed a balanced sampling of 3,068 genomes out of nearly 265,000 available on GISAID up to November 19th, 2020 (**Extended Data Table S1**), which ensured more accurate discrete phylogeographic reconstruction to unravel the likely origins of viruses from the 1st and 2nd infections. The genome sequences were aligned using MAFFT^58^, and the inference of the global phylogeny (n = 3,068 genomes) was performed using IQTree v.1.6.12, applying a GTR substitution model^59^, and TreeTime v.0.8.0^60^ within an augur pipeline^61^. From this large preliminary phylogeny (**Extended Fig. S1**), the expected clustering of the virus genomes sequenced from the patient was verified, and the sequence dataset was further subsampled, to include only 561 genomes belonging to closely related SARS-CoV-2 sublineages (**Extended** **Fig. S1**). The same approach implemented in the first round of analysis was used to generate the final phylogeny, but ultrafast bootstrap (1000 replicates)^62^ was performed to assess branch support. Phylogenetic data visualization was done using auspice (interactive view)^61^ and baltic 0.1.0 (static view). The search for polymorphisms in comparison with the reference genome (MN908947.3) was performed using Python scripts, and visualization of genome annotations were done using DNA Features Viewer 3.0.1^63^. The structure in Extended Data Fig. S2 (PDB 6VXX) was downloaded from PDB^51^, and formatted using PyMol. (The PyMOL Molecular Graphics System, Version 2.0 Schrödinger, LLC*.*)

*Flow cytometry*

As detailed in previous manuscripts and reproduced here for convenience^52^, antibody clones and vendors used for flow cytometry analysis were as follows: BB515 anti-hHLA-DR (G46-6) (1:400) (BD Biosciences), BV785 anti-hCD16 (3G8) (1:100) (BioLegend), PE-Cy7 anti-hCD14 (HCD14) (1:300) (BioLegend), BV605 anti-hCD3 (UCHT1) (1:300) (BioLegend), BV711 anti-hCD19 (SJ25C1) (1:300) (BD Biosciences), AlexaFluor647 anti-hCD1c (L161) (1:150) (BioLegend), biotin anti-hCD141 (M80) (1:150) (BioLegend), PE-Dazzle594 anti-hCD56 (HCD56) (1:300) (BioLegend), PE anti-hCD304 (12C2) (1:300) (BioLegend), APCFire750 anti-hCD11b (ICRF44) (1:100) (BioLegend), PerCP/Cy5.5 anti-hCD66b (G10F5) (1:200) (BD Biosciences), BV785 anti-hCD4 (SK3) (1:200) (BioLegend), APCFire750 or PE-Cy7 or BV711 anti-hCD8 (SK1) (1:200) (BioLegend), BV421 anti-hCCR7 (G043H7) (1:50) (BioLegend), AlexaFluor 700 anti-hCD45RA (HI100) (1:200) (BD Biosciences), PE anti-hPD1 (EH12.2H7) (1:200) (BioLegend), APC anti-hTIM3 (F38-2E2) (1:50) (BioLegend), BV711 anti-hCD38 (HIT2) (1:200) (BioLegend), BB700 anti-hCXCR5 (RF8B2) (1:50) (BD Biosciences), PE-Cy7 anti-hCD127 (HIL-7R-M21) (1:50) (BioLegend), PE-CF594 anti-hCD25 (BC96) (1:200) (BD Biosciences), BV711 anti-hCD127 (HIL-7R-M21) (1:50) (BD Biosciences), BV421 anti-hIL17a (N49-653) (1:100) (BD Biosciences), AlexaFluor 700 anti-hTNFa (MAb11) (1:100) (BioLegend), PE or APC/Fire750 anti-hIFNy (4S.B3) (1:60) (BioLegend), FITC anti-hGranzymeB (GB11) (1:200) (BioLegend), AlexaFluor 647 anti-hIL-4 (8D4-8) (1:100) (BioLegend), BB700 anti-hCD183/CXCR3 (1C6/CXCR3) (1:100) (BD Biosciences), PE-Cy7 anti-hIL-6 (MQ2-13A5) (1:50) (BioLegend), PE anti-hIL-2 (5344.111) (1:50) (BD Biosciences), BV785 anti-hCD19 (SJ25C1) (1:300) (BioLegend), BV421 anti-hCD138 (MI15) (1:300) (BioLegend), AlexaFluor700 anti-hCD20 (2H7) (1:200) (BioLegend), AlexaFluor 647 anti-hCD27 (M-T271) (1:350) (BioLegend), PE/Dazzle594 anti-hIgD (IA6-2) (1:400) (BioLegend), PE-Cy7 anti-hCD86 (IT2.2) (1:100) (BioLegend), APC/Fire750 anti-hIgM (MHM-88) (1:250) (BioLegend), BV605 anti-hCD24 (ML5) (1:200) (BioLegend), BV421 anti-hCD10 (HI10a) (1:200) (BioLegend), BV421 anti-CDh15 (SSEA-1) (1:200) (BioLegend), AlexaFluor 700 Streptavidin (1:300) (ThermoFisher), BV605 Streptavidin (1:300) (BioLegend). In brief, freshly isolated PBMCs were plated at 1–2 × 10^6^ cells per well in a 96-well U-bottom plate. Cells were resuspended in Live/Dead Fixable Aqua (ThermoFisher) for 20 min at 4 °C. Following a wash, cells were blocked with Human TruStan FcX (BioLegend) for 10 min at RT. Cocktails of desired staining antibodies were added directly to this mixture for 30 min at RT. For reinfection stains, cells were first washed and supernatant aspirated; then to each cell pellet a cocktail of secondary markers was added for 30 min at 4 °C. Prior to analysis, cells were washed and resuspended in 100 μl 4% PFA for 30 min at 4 °C. Following this incubation, cells were washed and prepared for analysis on an Attune NXT (ThermoFisher). Data were analysed using FlowJo software version 10.6 software (Tree Star). The specific sets of markers used to identify each subset of cells are summarized in Extended Data Fig. 9 of Lucas et al. (2020)^33^.

*SARS-CoV-2 specific-antibody measurements*

ELISAs were performed as previously described and reproduced here for convenience^52,64^. Briefly, Triton X-100 and RNase A were added to serum samples at final concentrations of 0.5% and 0.5mg/ml respectively and incubated at room temperature (RT) for 30 minutes before use to reduce risk from any potential virus in serum. 96-well MaxiSorp plates (Thermo Scientific #442404) were coated with 50 μl/well of recombinant SARS Cov-2 S1 protein (ACROBiosystems #S1N-C52H3-100ug) at a concentration of 2 μg/ml in PBS and were incubated overnight at 4 °C. The coating buffer was removed, and plates were incubated for 1h at RT with 200 μl of blocking solution (PBS with 0.1% Tween-20, 3% milk powder). Serum was diluted 1:50 in dilution solution (PBS with 0.1% Tween-20, 1% milk powder) and 100 μl of diluted serum was added for two hours at RT. Plates were washed three times with PBS-T (PBS with 0.1% Tween-20) and 50 μl of HRP anti-Human IgG Antibody (GenScript #A00166, 1:5,000) or anti-Human IgM-Peroxidase Antibody (Sigma-Aldrich #A6907, 1:5,000) diluted in dilution solution added to each well. After 1 h of incubation at RT, plates were washed three times with PBS-T. Plates were developed with 100 μl of TMB Substrate Reagent Set (BD Biosciences #555214) and the reaction was stopped after 15 min by the addition of 2 N sulfuric acid. Plates were then read at a wavelength of 450 nm and 570nm.

*Cytokine and chemokine measurements*

Patient serum was isolated as already described in this manuscript and previously reported^52^. Briefly, aliquots were stored at −80 °C. Sera were shipped to Eve Technologies (Calgary, Alberta, Canada) on dry ice, and levels of cytokines and chemokines were measured using the Human Cytokine Array/Chemokine Array 71-403 Plex Panel (HD71). All samples were measured upon the first thaw.

*Cell lines and virus*

As previously reported^65^, Vero E6 kidney epithelial cells were cultured in Dulbecco’s Modified Eagle Medium (DMEM) supplemented with 1% sodium pyruvate (NEAA) and 5% fetal bovine serum (FBS) at 37°C and 5% CO2. The cell line was obtained from the ATCC and has been tested negative for contamination with mycoplasma. SARS-CoV-2, strain USA-WA1/2020, was obtained from BEI Resources (#NR-52281) and was amplified in Vero E6 cells. Cells were infected at a MOI 0.01 for four three days to generate a working stock and after incubation the supernatant was clarified by centrifugation (450g × 5min) and filtered through a 0.45-micron filter. The pelleted virus was then resuspended in PBS then aliquoted for storage at − 80°C. Viral titers were measured by standard plaque assay using Vero E6 cells. All experiments were performed in a biosafety level 3 with the Yale Environmental Health and Safety office approval.

*Neutralization assay*

As reported in previous manuscripts and reiterated for convenience^65^, patients and healthy donor sera were isolated from whole blood and heat treated for 30 minutes at 56 °C. Sixfold serially diluted plasma, from 1:10 to 1:2430 were incubated with SARS-CoV-2 for 1 h at 37 °C. The mixture was subsequently incubated with Vero E6 cells in a 6-well plate for 1 hour for infection. Cells were overlayed with MEM supplemented NaHCO_3_, 2% FBS, and 0.6% Avicel mixture. Plaques were resolved at 40h post infection by fixing in 10% formaldehyde for 1h followed by staining in 0.5% crystal violet. All experiments were performed in parallel with healthy, uninfected control sera to establish degree of protection as reflected in difference in plaque counts.
